# Interaction between Per- and Polyfluorinated Substances (PFAS) and Acetaminophen in Disease Exacerbation—Focusing on Autism and the Gut–Liver–Brain Axis

**DOI:** 10.3390/toxics12010039

**Published:** 2024-01-03

**Authors:** Danielle Qiu Yun Jiang, Tai Liang Guo

**Affiliations:** Department of Veterinary Biomedical Sciences, University of Georgia, Athens, GA 30602, USA; danielle.jiang@uga.edu

**Keywords:** autism, PFAS, acetaminophen, gut–liver–brain axis, neurotoxicity, hepatoxicity, gut microbiome

## Abstract

This review presents a new perspective on the exacerbation of autism spectrum disorder (ASD) by per- and polyfluoroalkyl substances (PFAS) through the gut–liver–brain axis. We have summarized evidence reported on the involvement of the gut microbiome and liver inflammation that led to the onset and exacerbation of ASD symptoms. As PFAS are toxicants that particularly target liver, this review has comprehensively explored the possible interaction between PFAS and acetaminophen, another liver toxicant, as the chemicals of interest for future toxicology research. Our hypothesis is that, at acute dosages, acetaminophen has the ability to aggravate the impaired conditions of the PFAS-exposed liver, which would further exacerbate neurological symptoms such as lack of social communication and interest, and repetitive behaviors using mechanisms related to the gut–liver–brain axis. This review discusses their potential interactions in terms of the gut–liver–brain axis and signaling pathways that may contribute to neurological diseases.

## 1. Introduction

Autism, also known as autism spectrum disorder (ASD), is a neurodevelopmental disorder characterized broadly by challenges in social communication, repetitive behaviors, and restricted interests. Apart from the more widely acknowledged behavioral atypia associated with ASD, co-occurring conditions include psychological morbidity, e.g., anxiety, attention deficit/hyperactivity disorder (ADHD), intellectual disability, gastrointestinal signs, and insomnia [1]. In 2020, the U.S. Centers for Disease Control and Prevention’s (CDC) Autism and Developmental Disabilities Monitoring (ADDM) Network found that 1 in 36 children below the age of eight was diagnosed with ASD across eleven states in the United States [2]. This prevalence represents the highest of all surveillance years since 2000, and possibly suggests a nation-wide phenomenon of autism spike.

Rapid advancement in technology and scientific research facilitated a more in-depth exploration of the mechanisms underpinning the autistic phenotype. In the past, genetic factors and pathoanatomic brain defects were well studied as the causality of autism and other neurological diseases in children. The drive to identify brain-centric disease mechanisms overshadowed the exploration of less direct, but nonetheless potentially important, contributors to ASD development, such as pharmaceuticals, lifestyle habits, environmental pollutants including exposure to pesticides, and immune system disorders. Areas of recent research, such as the impacts of selective drugs and environmental toxins, were intensively pursued in the past decade to better understand other contributors to ASD development [3,4,5,6,7,8]. Similarly, contemporary cross-disciplinary (immunology, metabolomics, and neuroscience) studies have revealed dynamic interactions between the host immunity and gut bacterial communities that may contribute to neurological diseases [9,10,11,12,13,14,15].

This review systematically discusses evidence reported on the involvement of the gut microbiome and liver inflammation that may lead to the onset of ASD symptoms, e.g., the gut–liver–brain axis. In addition, we summarize pertinent literature on the potential mechanisms of per- and polyfluoroalkyl substances (PFAS)-induced toxicity in the gut, liver, and brain. The potential contribution and interaction of both PFAS and acetaminophen to the onset of clinical neurologic disease, particularly autism, were also evaluated. Cross-disciplinary approaches, including those in neuroscience, toxicology, physiology, and immunology, have been used to critically assess various contributions to disease progression or exacerbation. In conducting this review, various databases including Google Scholar and PubMed were searched using terms such as autism, inflammation, acetaminophen, glutathione, PFAS, neurotoxicity, hepatoxicity, and microbiome.

## 2. The Gut–Liver–Brain Axis

**Figure 1:**
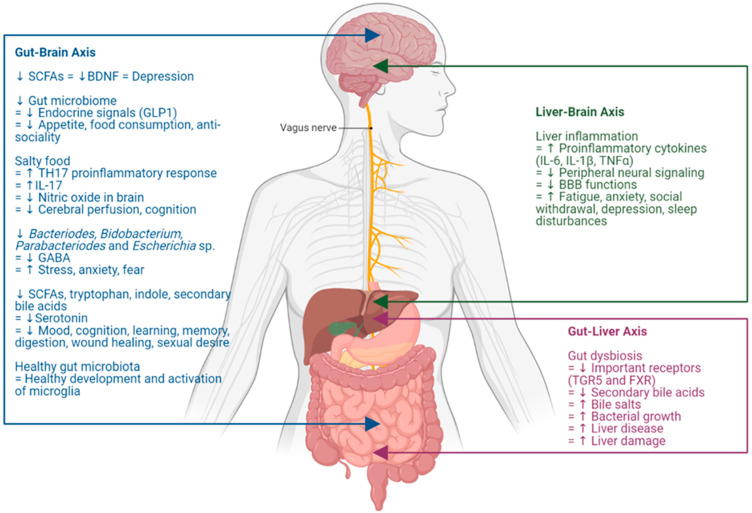
Gut–liver–brain axis. A summary of the interactions within the gut–liver–brain axis that contribute to neurological diseases and symptoms. Gut–brain axis (blue). Liver–brain axis (green). Gut–liver axis (red). Abbreviations: ↑ indicates an increase in the condition or level; ↓ indicates a decrease in the condition or level; = indicates a “lead to the condition or level”; BBB, blood brain barrier; BDNF, brain-derived neurotrophic factor; FXR, farnesoid-X-receptor; GABA, gamma-aminobutyric acid; GLP, glucagon-like peptide; IL, interleukin; SCFAs, short-chain fatty acids; TGR5, Takeda G protein-coupled receptor 5; TH, T helper; TNF-α, tumor necrosis factor alpha. Created with Biorender.com, accessed on 10 September 2023.

The gut–liver–brain axis constitutes a multidirectional communication network that connects the enteric, hepatic, and central nervous systems (Figure 1). Through the complex interplay between the gut–liver, gut–brain, and liver–brain axes, this communication network extends to involve endocrine, immune (humoral), and metabolic routes of communication. Within the network, the gut and liver affect cognitive behaviors through the host’s immune responses and the regulation of microbiota, and the brain also influences intestinal and hepatic activities. Studies in animals have shown that an impaired gut–liver–brain axis is associated with diseases such as hepatic encephalopathy, Alzheimer’s disease, Parkinson’s disease, Multiple Sclerosis, depression, and ASD [16,17,18,19,20,21,22,23,24].

### 2.1. Gut–Brain Interactions

The gut microbiota modulates gastrointestinal homeostasis in experimental animals through direct and indirect chemical signaling with the nervous system [25,26]. An example of direct signaling is the regulated expression of brain-derived neurotrophic factor (BDNF), a neuronal factor associated with depression, by short-chain fatty acids (SCFAs) produced in the gut [27]. SCFAs are lipids produced by the gut microbiome that can influence the central nervous system (CNS) through the regulation of the immune system, neuroplasticity, expression of various genes, and epigenetic changes [27]. The gut microbiome can also influence the host’s appetite, feeding behaviors, and digestion through indirect chemical signaling. For example, within gut epithelium, the microbiota can regulate the production of endocrine signals, such as the hormone glucagon-like peptide 1 (GLP-1), from the enteroendocrine cells [28]. As such, germ-free (GF) mice that lack an endogenous microbiota have lower food consumption as compared to conventional mice with intact microbiota [29]. In addition, the gut microbiota has been related to the typical ASD behaviors in mice. For example, GF mice exhibit anti-sociality and prefer to spend time exploring an empty compartment as compared to where another mouse companion is present [30]. Diets play a part in brain health as well. Foods high in sodium trigger a proinflammatory response in the intestine, e.g., increased secretion by T helper 17 (TH17) cells of the proinflammatory cytokine interleukin-17 (IL-17) into the bloodstream. IL-17, in turn, inhibits the production of nitric oxide by neuroparenchymal vascular endothelial cells, impairing cerebral perfusion and thus cognition [31].

The gut microbiota also modulates the production and synthesis of neurotransmitters in the hosts. For example, in silico and in vitro studies [32,33] have shown that microorganisms such as Bacteriodes, Bifidobacterium, Parabacteriodes, and Escherichia spp. can produce γ-aminobutyric acid (GABA), a neurotransmitter that regulates neuronal cell hyperactivity associated with stress, anxiety, and fear [34]. In vivo studies in rats using Bifidobacterium strains from humans [32] and cell culture studies [35] have shown an upregulated expression of GABA. However, it has not been demonstrated that the GABA produced by Bifidobacterium is resorbed from the gut and circulates in the body to affect the brain. In male GF mice, gut bacteria, through interacting with the enteroendocrine cells, play a vital role in the production of serotonin (5-hydroxytryptamine), a neurotransmitter that regulates body functions such as mood, cognition, learning, reward, memory, digestion, wound healing, and sexual desire [11]. The production of serotonin can be affected by microbial metabolites such as SCFAs, tryptophan, indole, and secondary bile acids [36,37]. It is also important to note that most neurotransmitters produced by the microbiota, such as serotonin, aminobutyric acid, and dopamine, cannot reach the brain directly due to the blood–brain barrier. However, in rats [38], neurotransmitters can cross the blood–brain barrier indirectly through neurotransmitter precursors, such as tryptophan (serotonin precursor), before being converted into active neurotransmitters [39,40,41]. The mechanisms of neurotransmitters produced from the gut microbiota to influence the functions of other body parts have not been well-established, presenting an avenue for future research focusing on the interactions between the gut and brain. It was hypothesized that, in the gut, tryptophan undergoes three major metabolic pathways, e.g., the 5-HT, kynurenine, and AhR ligand pathways, which may be directly or indirectly controlled by saprophytic flora [42].

The major neuronal pathway facilitating gut–brain interactions is the vagus nerve that extends from the brainstem to innervate both the gut and enteric nervous system [43]. Influenced by the gut microbiota, the enteroendocrine cells produce chemical stimuli, such as neurotransmitters, hormones, and metabolites, to trigger the production of chemoreceptors that activate mechanoreceptors to relay signals from the vagus nerve to the CNS [44]. Neurotransmitters, integral to the gut–brain interactions, can be produced by the gut microbiota through the metabolism of indigestible fibers such as cellulose, lignin, beta-glucans, and pectin. Specifically, dopamine and norepinephrine are produced by members of the Bacillus family; GABA by the Bifidobacteria family; GABA and acetylcholine by the Lactobacilli family; norepinephrine and serotonin by the Escherichia family; and serotonin by the Enterococcus and Streptococcus families [13]. In addition, Bacteroides spp. regulate the development of enteric cells in mice, which play important roles in the maintenance of neuronal networks and regulation of gut homeostasis [9,45]. Healthy development and activation of microglia, the innate immune cells of the brain, are likewise modulated by microbiota. In separate studies, GF mice treated with SCFAs and Bifidobacterium spp. exhibited restoration of microglial morphology and functions [12,14].

### 2.2. Gut–Liver Interactions

Gut dysbiosis can contribute to metabolic disorders in the liver of both humans and experimental animals, such as alcoholic and non-alcoholic fatty liver disease (NAFLD), nonalcoholic steatohepatitis, primary sclerosing cholangitis, cholestatic liver disease, hepatocellular carcinoma, and cirrhosis [46,47,48,49,50,51,52,53]. In the bidirectional relationship, communication with each other is connected through the portal vein, biliary tract, and systemic circulation in humans. From the intestine, microbial metabolites are transported to the liver through the portal vein. Meanwhile, to maintain gut eubiosis and control bacterial overgrowth, bile salts and antimicrobial molecules are transported from the liver to the intestinal lumen through the biliary tract [54,55,56].

In mice, an impaired synergistic relationship within the gut microbiota can lead to gut dysbiosis and reduce the activation of important receptors such as membrane G protein-coupled receptor TGR5 and nuclear bile acid receptor FXR. Such impairment can lead to a decrease in secondary bile acids synthesis, followed by the retention of bile salt, bacterial overgrowth, and liver disease that may potentially progress to liver failure [57,58]. One example would be hepatic encephalopathy, a typical disease model of a dysregulated gut–liver–brain axis. Symptoms from hepatic encephalopathy can be alleviated by improving the axis via treatment with Lactobacillus in mice [59] and fecal translocation in mice with steatohepatitis [60,61]. It was recently postulated that in mice and humans, a sustained damage to the inner gut vascular barrier in the gastrointestinal tract is a key player along the gut–liver–brain axis, as it has the ability to influence beyond the liver to distal organs including the brain [62].

### 2.3. Liver–Brain Interactions

Hepatic dysfunction can lead to CNS dysfunction through alterations in CNS blood flow, the presence of inflammatory metabolites, excess bile acids, and accumulation of neurotoxic compounds such as ammonia in mice [63,64,65]. In patients with chronic liver conditions, neurological symptoms such as fatigue, anxiety, social withdrawal, depression, and sleep disturbance have been observed [66]. It has been recently shown that potential mechanistic avenues within the gut–liver–brain axis may be altered in the setting of chronic liver diseases, which subsequently contribute to the neurological disorders mentioned above [67,68,69,70,71,72].

Cytokine-mediated signaling is thought to affect the neurotransmission within the basal ganglia and cause CNS dysfunction. In the setting of intrahepatic inflammation, liver immune cells produce proinflammatory cytokines such as IL-6, IL-1β, and tumor necrosis factor (TNF)-α. These inflammatory cytokines can induce neurological changes by affecting the peripheral neural signaling; they can also enter the CNS through systemic circulation and the disrupted blood–brain barrier to affect the neurons within [73].

The vagus nerve, as mentioned in the section on gut–brain interactions, is the major neuronal pathway for the communication between the gastrointestinal tract and CNS. The vagus nerve is bilateral, with the left and right nerves in part having distinct functions. In several studies, the left vagus nerve has been shown to carry the signals from the liver to the brain [74]. Through this pathway, recent work has portrayed a new neuroimmune pathway in which the liver has demonstrated gut-dependent sensing and signaling to promote an anti-inflammatory state through the brain. Upon the sensing of luminal contents in the gastrointestinal tract, the liver afferent vagal fibers transmit sensory inputs to the nucleus tractus solitarius of the brainstem to induce and maintain gut T-regulatory cells through enteric neurons and parasympathetic nerve signaling [75].

## 3. Polyfluoroalkyl Substances

In the late 1930s, PFAS were discovered, and many products commonly used by consumers and industry have been manufactured with or from PFAS since the 1950s. Known as the “forever chemicals”, PFAS comprise a heterogenous group of nearly 15,000 synthetic chemicals that have, since a decade ago, been of concern to regulatory authorities due to their persistence and wide-spread accumulation in the environment [76]. Due to their thermal stability, and hydrophobic and oleophobic properties, PFAS are widely applied in consumer and industrial products such as non-stick cookware, paper, food packaging, carpets, metal plating processes, and aqueous film forming foams for firefighting [77]. Within this broad class of chemicals, perfluorooctanoic sulfonate (PFOS) and perfluorooctanoic acid (PFOA) garnered the attention of regulatory bodies and research institutions due to their wide distribution in aquatic environments, and potential accumulation and toxicity in humans through biomagnification via food webs [78].

Since 1999, the National Health and Nutrition Examination Survey (NHANES) has surveyed the blood PFAS levels in volunteers from the U.S. population biyearly. Because of regulatory restrictions on the production and use of PFOS, PFOA, perfluorohexane sulfonic acid (PFHxS), and perfluorononanoic acid (PFNA) within the U.S., blood levels for PFOS and PFOA had declined significantly by 85% and 70%, respectively, from 1999 to 2018 [79]. Such biomonitoring studies included workers in PFAS manufacturing facilities and communities with contaminated drinking water. As PFAS accumulates in the body, exposure assessments and studies were also conducted to understand their adverse effects on human health. Elevated levels of PFAS are associated with adverse effects on growth development and thyroid, reproductive, immune, and hepatic functions. However, the toxicity studies remain insufficient in many areas, especially in the field of neurotoxicity. Thus, the immediate priority in this emerging area is to conduct risk exposure assessments and develop quantification methods on the thousands of untested PFAS. Since limited biological mechanisms of PFAS action within target tissues, e.g., endocrine disruption, are known, this review attempted to summarize the gut–liver–brain axis as a potential pathway for their neurotoxic effects.

### 3.1. Liver Inflammation and Polyfluoroalkyl Substances

Liver toxicity is a hallmark of PFAS. Exposure studies in rodents found liver enlargement, elevated liver enzymes, hepatocellular hypertrophy, and hepatic steatosis (lipid accumulation) (Table 1). PFAS exposure also significantly correlated with decreased insulin resistance, increased liver fat content, and enhanced histological liver lesions [80]. Although the full spectrum of mechanisms of PFAS hepatoxicity remain undetermined, one potential pathway is through lipid disruption and inducing NAFLD. Studies have found a strong association between alanine aminotransferase (ALT), the liver enzyme marker for NAFLD, and the level of serum PFAS [81,82,83]. Other liver function biomarkers, such as aspartate aminotransferase (AST), gamma-glutamyltransferase (GGT), and alkaline phosphatase (ALP), were found to be positively associated with plasma concentrations of PFOS, PFOA, PFHxS, and PFNA in humans [82,83].

It had been proposed that PFAS dysregulate hepatic lipid metabolism and promote liver inflammation by activating the peroxisome proliferator-activated receptor alpha (PPARα) in both humans and mice [84,85]. However, others debated that the liver injury caused by PFAS may not depend on PPARα alone, as complementary mechanisms involving other receptors could be at play, such as the constitutive androstane receptor (CAR) and the pregnane X receptor (PXR). Through the modulation of cell signaling, PFAS also contribute to cell apoptosis by increasing reactive oxygen species (ROS) production and reducing the Nrf2-regulated antioxidant defense system [86]. A recent exploration of the pathogenic mechanism using human hepatocyte cell line HepG2 revealed a linkage between PFOA exposure and the derangement of hepatocyte cell metabolism. It was found that PFOA exposure caused significant impairment of the insulin receptor (InsR) signaling pathway, which resulted in downstream altered glycogen synthesis and reduced glucose uptake [87]. Similarly, HepaRG, a surrogate for primary human hepatocytes, and HepG2 cells treated with PFOA, heptafluorobutyric acid (HFBA), and perfluorotetradecanoic acid (PFTA) showed increases in: (1) ROS production; (2) the expression of inflammatory markers such as pro-inflammatory cytokine TNF-α and pro- and anti-inflammatory myokine IL-6; (3) the expression of unfolded protein response signaling pathway markers such as IRE1α, ATF4, and BIP; (4) the expression of fatty acid metabolic gene markers such as stearoyl-CoA desaturase-1 (SCD1), fatty acid synthase (FASN), acetyl-CoA carboxylase (ACC), and transcription factors including sterol regulatory element-binding protein 1 (SREBP1); (5) the expression of fibrosis signaling gene markers such as TIMP2, p21, TGFβ, and finally; (6) the expression of genes associated with NAFLD [86,88]. Other PFAS exposure studies on HepG2 cells found a decrease in glutathione levels [89].

**Table 1:** Studies reporting the liver toxicity effects of PFAS exposure.

Organism	Sample Type	Sample Size	Age	Sex	Reagents	Exposure Dose	Exposure Time	Outcome	References
Humans (Cross-sectional)	Liver	105	18 to 75	M, F	PFAS	Population study	Environmental/ Lifestyle	↓ Insulin resistance ↑ Liver fat content ↑ Histological liver lesions	[80]
Humans (Cross-sectional)	Blood serum	46,452	Adults	M, F	PFOS PFOA	Median (IQR) PFOA: 28.0 (13.5–70.8) ng/mL PFOS: 20.3 (13.7–29.4) ng/mL	2005–2006	↑ Alanine aminotransferase (ALT)	[81]
Humans (Cross-sectional)	Plasma serum	230 to 2288	20 to 74	M, F	PFOS PFOA PFHxS PFNA	Population study	Environmental/ Lifestyle	↑ Aspartate aminotransferase (AST) ↑ Gamma-glutamyltransferase (GGT) ↑ Alkaline phosphatase (ALP)	[82]
Humans (Retrospective)	Blood serum	2883	Adults (obesed)	M, F	PFOS PFOA PFHxS PFNA	Geometric mean (95% CI) PFOA: 2.0 (1.8–2.1) ng/mL PFOS: 5.5 (5.0–6.0) ng/mL PFNA: 0.73 (0.68–0.79) ng/mL PFHxSe: 1.24 (1.13–1.37) ng/mL	2011–2014	PFOA, PFHxS and PFNA: ↑ Alanine aminotransferase (ALT) PFOA and PFNA: ↑ Gamma-glutamyltransferase (GGT)	[83]
Rats	Plasma	90 (3 doses × 3 expsoure period × 10/dose group)	6–7 weeks	M	K^+^PFOS	20 and 100 ppm	1, 7 and 28 days	↑ Persoxisome proliferator-activated receptor alpha (PPARa) ↑ Constitutive androstane receptor (CAR) ↑ Pregnane X receptor (PXR)	[85]
HepaRG and HepG2 cells	Liver cells	Not indicated	Not relevant	Not relevant	PFOA HFBA PFTA	5 to 1000 μM	24 h to 10 days	↑ Cell apoptosis ↑ ROS production ↑ Pro-inflammatory cytokine (TNFα, IL6) ↑ UPR signalling pathway markers (IRE1α, ATF4, BIP) ↑ Fatty acid metabolic gene markers (SCD1, FASN, ACC) ↑ Transcription factors (SREBP1) ↑ Fibrosis signalling gene markers (TIMP2, p21, TGFβ) ↓ Nrf2 regulated antioxidant defense system	[86]
HepG2 cells	Liver cells	Not indicated	Not relevant	Not relevant	PFOA	0, 0.1, 1, 10, 100 and 1000 ng/mL	24 h	↑ Impairment of insulin receptor (InsR) signalling pathway ↑ Altered glycogen synthesis ↓ Glucose uptake	[87]
Humans (Cross-sectional)	Blood serum	74	Children (with NAFLD)	M, F	PFOS PFOA PFHxS	Median (IQR) PFOA: 3.42 (1.65) ng/mL PFOS: 3.59 (4:46) ng/mL PFHxS: 1.53 (3:17) ng/mL	2007–2015	↑ NAFLD ↑ NASH ↑ Fibrosis ↑ Lobular/portal inflammation ↑ NAFLD activity scire	[88]
HepG2 cells	Liver cells	Not indicated	Not relevant	Not relevant	PFOA PFOS PFNA PFDA PFHxS	0.2, 2 and 20 μM	24 h	↓ Glutathione levels	[89]

Abbreviations: ↑ indicates an increase in the condition or level; ↓ indicates a decrease in the condition or level.

### 3.2. Neuroinflammation and Polyfluoroalkyl Substances

Based on several recent epidemiology surveillance studies, no consistent evidence of an increased risk between PFAS exposure and neurotoxicity had been observed [90,91]. However, in-depth studies at cellular and molecular levels (Table 2) have shown that PFAS possess the potential to trigger and/or participate in pathways that may lead to neurobehavioral disorders, such as ADHD, fetal congenital cerebral palsy, memory dysfunction, learning disorders, and intellectual disability. Similarly, the identification of key events at both the cellular and molecular levels is crucial in developing potential preventative and therapeutic strategies to manage neurological disorders.

Between ADHD and ASD, it is important to note that, although both have distinct diagnostic criteria, they often occur concurrently. It was reported that youths with ASD exhibit comorbidity rates with 71% having ADHD, whereas 12.4% of children with ADHD were reported to display ASD traits [92]. Prevalence studies examining the relationship between PFAS and ADHD had shown conflicting results with associations ranging from negative to positive. In areas where children drank PFOA-contaminated water, studies showed a negative association between PFOA exposure and ADHD in children. Conversely, a birth cohort study in Norway found a positive correlation between PFOS concentration in breast milk and the child developing ADHD by the age of thirteen. Studies with both large and small sample sizes (*n* = 59–4826) found no correlation between prenatal exposure of PFOS and PFOA and ADHD, although there were differing sex-dependent results where there was a positive association of PFAS exposure and ADHD in female infants [93]. Other sex-specific studies found positive associations between the serum PFOA level and ADHD in only boys, and positive correlations between the concentrations of PFOA, PFOS, and perfluoroheptane sulfonic acid (PFHpS) in maternal plasma and the risk of cerebral palsy in only male infants also [93]. These studies, however, showed limitations in the sample size, inconsistent results, and a lack of defining sex-related mechanisms.

As compared to the blood and liver, the brain is not a dominant tissue for PFAS accumulation. Within the brain, higher PFAS levels were found in the brain stem, thalamus, hypothalamus, hippocampus, and pons/medulla due to their close proximity to the incoming bloodstream [93]. Based on previous studies, it was proposed that PFAS entered the brain by disrupting the tight junctions of the blood–brain barrier, or by binding to the transporters located at the blood–brain barrier to facilitate transport through the endothelial cell membrane. However, these studies used renal transporters as models and not that of the blood–brain barrier [93]. While several potential mechanisms of PFAS-induced neurotoxicity have been proposed, only three (i.e., calcium homeostasis, calcium-dependent signaling molecules, and neurotransmitters) have received considerable attention thus far. These three mechanisms are further explained as follows.

Calcium dyshomeostasis: Within laboratory-cultured rat hippocampal neurons, PFOA and PFOS may significantly increase intracellular calcium concentrations due to the release of calcium from the mitochondria and endoplasmic reticulum. Excessive intracellular calcium concentrations might then potentiate synaptic transmission, provoke neuronal excitement, induce oxidative stress events and, eventually, lead to neuronal dysfunction and apoptosis. In addition, the magnitude of calcium release appeared to be age dependent as PFOS exposure incited calcium release in the brain microsomes of adult rats, but not neonatal rats [93,94].

Altered expression of calcium-dependent signaling molecules: PFAS altered the expression of calcium-dependent signaling molecules in rat neurons within the cerebral cortex (PFOS), hippocampus (PFOS and PFOA), and cerebellar Purkinje cells [94,95,96]. Affected calcium-dependent signaling molecules comprise the Ca^2+^/calmodulin-dependent protein kinase II (CaMKII), cAMP-response element binding protein (CREB), and calcineurin (CaM). These molecules are critical for preserving neuronal functional and structural integrity, and facilitate learning, memory, and cognition. Similarly, after prenatal exposure to PFOS and PFOA, proteins critical for growth (GAP-43), synaptogenesis (synaptophysin), and neuronal development (tau) were increased in the cortex and hippocampus of the mice. Such alteration and overexpression of molecules and proteins could result in oxidative stress, which eventually lead to cell apoptosis and behavioral deficits, such as ADHD and response inhibition [93].

Dysregulation of neurotransmitters: PFAS cause the dysregulation of neurotransmitters, which consist mainly of dopamine and glutamate, and acetylcholine of the cholinergic system. Such neurotransmitters are neuron-generated chemicals that play an essential role in signal transmission. In various exposure studies, PFOS and PFOA were observed to alter the level of dopamine in the brains of mice, rats, and frogs [96,97,98,99,100]. In high-throughput-targeted metabolomics studies, PFOA increased dopamine concentrations in male mice [100]. Another study investigating different brain regions showed that PFOS increased dopamine concentrations in the prefrontal cortex and hippocampus of adult mice [99]. However, in Northern leopard frogs, PFOS and PFOA decreased dopamine concentration. It was suggested that the frogs could be a more suitable test model than rodents for the study of Parkinson’s disease, due to the presence of neuromelanin-containing dopaminergic neurons in the brain [97]. Glutamate, another neurotransmitter for memory and learning, was increased in Northern leopard frogs and the hippocampus of adult mice after PFOS exposure [97,101]. On the contrary, glutamate was decreased after PFOA exposure in mice [100]. Another mechanism related to neurotransmitters was an altered expression of dopamine receptors, such as the decrease in dopamine receptor-D5 in the mouse cerebral cortex and dopamine receptor-D2 in the mouse hippocampus after PFOS exposure [98]. In addition, PFOS and PFOA were also found to damage the cholinergic systems of adult mice at low dose exposures [102].

### 3.3. Other Neurological Diseases and Polyfluoroalkyl Substances

PFAS potentially increase inflammasome activation in the brain, possibly causing synuclein aggregation and dopaminergic degeneration as has been demonstrated in Parkinson’s disease patients. Though the mechanisms of dopaminergic degeneration through PFAS exposure have not been deeply investigated, inflammasome activation is a potential therapeutic target for Parkinson’s disease. It was proposed that the inflammasomes could activate NOD-, LRR-, and pyrin domain-containing protein 3 (NLRP3) upon sensing dysbiosis within the gut microbiome and host immunometabolic disruption induced by toxicants, and eventually lead to neuronal dysfunction [24,103,104]. Again, this is an unexplored area that could provide important insights into the gut–brain axis of people with Parkinson’s disease.

In Italy, an ecological study was conducted to compare the mortality causes of death in municipalities with PFAS-contaminated and -uncontaminated drinking water from 1980 to 2013. In contaminated municipalities, both sexes showed statistically significant relative risks for cerebrovascular diseases and Alzheimer’s diseases. In addition, females were found to have significant relative risk for Parkinson’s disease [105].

An interesting short study was conducted to find the association between prenatal exposure to PFAS and facial features of 5-year-old children in Denmark. With a small sample size of 656 children, prenatal exposure to PFAS was found to be associated with shorter palpebral fissure length, which is a distinct facial characteristic of down syndrome. The children with shorter palpebral fissure length also had lower IQ scores and behavioral impairment [106]. The study of craniofacial development is felt by many to be an understudied area that may provide potential links to neurological assessment and brain development. For example, exposure to exogenous chemicals such as PFAS could disrupt the developmental processes of neural crest cells that would develop into neuroglia and craniofacial cartilage. Similarly, children with orofacial cleft are also associated with higher probabilities of language disorders, intellectual disability, and psychiatric disorders [107].

### 3.4. Gut Microbiome and Polyfluoroalkyl Substances

The gut microbiome comprises a heterogenous population of microorganisms (bacteria, fungi, viruses, and archaea) that reside within the gastrointestinal tract. Broadly, functional gut microbiota facilitate digestion, detoxification, and production of nutrients, maintain the structural integrity of the gut mucosal barrier, regulate the immune system, and protect against enterotropic pathogens [108]. More specifically, the gut microbiome plays relevant roles in the aggravation and alleviation of PFAS toxicity. Through altering the intestinal barrier and gut environment, PFAS could modify the microbiota composition, affect its synthesis of certain vitamins and amino acids, and secondarily impact metabolic pathways. The ability of the gut microbiome to tolerate and respond to the presence of toxicants within the gut ecosystem might play a key role in the seemingly widely variable responses observed across multiple studies.

Several studies have evaluated the relationship between PFOS and the intestinal microbiota. In summary (Table 3), the studies found that PFOS induced decreases in: (1) phylum: Bacteroidetes and Firmicutes, (2) class: Clostridial and Gammaproteobacteria, (3) family: Erysipelotrichaceae and Enterobacterial; and (4) genus: Alistipes, Blautia, Faecalibacterium, Flavonifractor, Ihubacter, Lactobacillus, Legionellales, Ligilactobacillus, Limosilactobacillus, Neglecta, Parasutterella, Stigonematales, and Thermogemmatisporales. For other gut bacteria, increases were found in: (1) phylum: Bacteroidetes and Proteobacteria; (2) family: Rikenellaceae and Ruminococcaceae; (3) genus: Bifidobacterium, Bilophila, Clostridium, Escherichia/Shigella, Gemella, Parabacteroides, Streptococcus, and Turicibacter; and (4) species: Bilophila wadsworthia, Faecalibacterium prautzii, Dorea longicatena, and Sutterella wadsworthensis in male mice. PFOS also disturbed the biosynthesis of flavonoid and steroid hormones, reducing the levels of both SCFAs and occludin, a tight junction protein [10,109,110,111,112,113,114,115].

For PFOA, the abundances of Phyla Bacteroidetes and species Odoribacter splanchnicus were increased, accompanied by a decrease in genus: Akkermansia, Anoxybacillus, Bifidobacterium, Gemmiger, Parabacteroides, and Ruminococuscus [114,116]. The male mice also suffered a loss in intestinal barrier integrity, decreased concentrations of SCFAs, and a reduced capability in spatial memory and learning. SCFAs, e.g., propionate and butyrate, can promote the production of essential neurotransmitters of the gut–brain axis through modulating peptide YY release and enteroendocrine serotonin secretion [111]. In terms of neuroinflammation, PFOA exposure caused an increase in the LPS content and TNF-α levels in the mouse cortex, which led to cognitive deficits and dysbiosis with the gut and brain. Fecal microbiota transplantation treatment was then applied to the same group of mice, which relieved and mitigated the symptoms. That study demonstrated that microbiota played separate roles in the aggravation and alleviation of neurotoxicity [10,114,116].

Prenatal exposure to PFOS and PFOA also modified the microbiome. High PFAS in maternal blood could significantly decrease the abundance of Faecalibacterium in the meconium and increase the abundance of genus, e.g., Bifidobacterium, Gemella, Staphylococcus, and Clostridium spp., in fecal samples [111,112]. In the breast milk of exposed mothers, the concentrations of PFOS and PFOA were associated with an increase in pathogenic Enterococcus that was linked to infection, and with a decrease in microbiome α-diversity and Lactobacillus that are important immune modulators [110]. One recent study explored the relationship between indoor PFAS exposure and the gut microbiomes of children in the U.S. The results showed that there was a negative association between PFOS and the abundance of beneficial taxa that play critical roles in nutrient absorption, such as Legionellales, Stigonematales, and Thermogemmatisporales at the order level [105,107]. While confounding factors, e.g., types of food consumed, are considered in PFAS intake, no studies have been conducted on this aspect. However, Iszatt et al. [110] made an observation that people from countries with lower environmental controls were more susceptible to PFAS exposure due to the contaminated fish and meat.

**Table 2:** Studies reporting the neurotoxicity effects of PFAS exposure.

Organism	Sample Type	Sample Size	Age	Sex	Reagents	Exposure Dose	Exposure Time	Disease/Disorder	Outcome	References
Rats	Brain (hippocamal neurons)	Not indicated	Not indicated	Not indicated	PFOA PFOS	30, 100 and 300 µmol/L	30 min	ADHD	↑ Calcium concentration in neurons ↑ Synaptic transmission ↑ Neuronal excitement ↑ Cell apoptosis	[94]
Rats	Brain, (cortex and hippocampus)	4 groups of 8 to 10	Adult	M	PFOS	1.7, 5.0, and 15.0 mg/L	91 days	ADHD	↑ Alteration of calcium-dependent signalling molecules expression (CaMKII, CREB, CaM)	[96]
Northern leopard frogs	Whole body	Not indicated	Larvae	M, F	PFOA PFOS	10, 100, and 1000 ppb	30 days	ADHD	↓ Dopamine ↑ Glutamate	[97]
Rats	Brain (amgydala, prefrontal cortex and hippocampus)	5 groups of 6	2 months	M	PFOS	0.5; 1.0; 3.0 and 6.0 mg/kg	28 days	ADHD	↑ Dopamine	[97,99]
Mice	Brain (cerebral cortex and hippocampus)	Not indicated	10 days	M	PFOS	11.3 mg/kg	24 h; 2 months post exposure	ADHD	↓ Dopamine receptor-D5 ↓ Dopamine receptor-D2	[98]
Mice	Brain and liver	Not indicated	Not indicated	M	PFOA PFAS mixtures	0.5 and 2.5 mg/kg	28 days	ADHD	Dopamine concentration:↑ PFOA	[100]
Mice	Brain and liver	Not indicated	Not indicated	M	PFOA	2.5 mg/kg	28 days	Impaired neurodevelopment	↓ Glutamate	[100]
Mice	Brain (hippocampus)	4 group of 15	2 months	M, F	PFOS	10.75 mg/kg	3 months	Impaired spatial learning and memory	↑ Glutamate	[101]
Mice	Behaviourial test	Not indicated	Neonatal	M	PFOA PFOS	PFOA: 0.58 or 8.70 mg/kg PFOS: 0.75 or 11.3 mg/kg	10 days	Associated with neurodevelopment	↓ Cholinergic system	[102]
Mice	Lung	3 group of 8	Pregnant adults	F	PFOS	0, 1 or 5 mg/kg	Gestational day 12 to 18	Parkinson’s disease	↑ Inflammasome activation in the brain ↑ NOD-, LRR- and pyrin domain-containing protein 3 (NLRP3)	[103]
Mice	Dopaminergic primary cultured neurons	2 groups of 6	2 months	M	PFOS	10 mg/kg	14 days	Parkinson’s disease	↑ Synuclein aggregation ↑ Dopaminergic degeneration	[104]
Humans	Epidemiological database	369,826	0 to > 74	M, F	PFAS	Ecological mortality study	1980 to 2013	Cerebrovascular diseases Alzheimer’s diseases Parkinson’s disease	Ecological mortality study ↑ Significant relative risk	[105]
Humans	Maternal plasma and fetal physical features	656	5	M, F	PFAS	Birth Cohort Study	Prenatal exposure	Down syndrome	Shorter palpebral fissure length Lower IQ scores Behavioural impairment	[106]
Mice	Brain, liver, intestines, blood and faeces	5 groups of 10	8 weeks	M	PFOA	0, 0.5, 1, and 3 mg/kg	35 days	Associated with brain inflammation and impairment	↑ LPS content and TNF- α levels in the cortex ↑ Cognitive deficits and dysbiosis with the gut and brain	[116]
Humans	Plasma serum and cord blood	725	Maternal and fetal	M, F	PFHxS	Birth Cohort Study	Prenatal exposure	Associated with fetal neurodevelopment	↑ Brain-derived neurotrophic factor (BDNF) expression	[117]

Abbreviations: ↑ indicates an increase in the condition or level; ↓ indicates a decrease in the condition or level.

**Table 3:** Studies reporting the effects of PFAS exposure on gut microbiome.

Organism	Sample Type	Sample Size	Age	Sex	Reagents	Exposure Dose	Exposure Time	Outcome	References
Human	Blood, urine and faeces	79	Nil	M, F	PFAS	Population study	Nil	↓ genus: *Thermogemmatisporales*, *Stigonematales*, and *Legionellales*	[109]
Humans	Breast milk and infant faeces	Breastmilk: 333 mothers and 328 children Faeces: 535 mothers and 552 children	Not indicated	M, F	PFOS PFOA	Cohort study PFOS: 0.12 ng/mL PFOA: 0.05 ng/mL	2002 to 2005	↓ microbiome α-diversity and *Lactobacillus* ↑ *Enterococcus* in breast milk	[110]
Human	Maternal blood, cord blood and and infant faeces	Nil	Nil	M, F	PFOS PFOA	Cohort study Maternal blood: 2.4 ng/mL Cord blood: 1.14 ng/mL	Nil	↓ genus: *Faecalibacterium* ↑ genus: *Clostridium*, *Streptococcus*, *Gemella* and *Bifidobacterium*	[111,112]
Mice	Liver, faeces	8 groups of 6	2 months	M	PFOS	5, 10 and 20 mg/kg	14 days	↓ genus: *Lactobacillus*, *Limosilactobacillus*, *Neglecta*, *Ligilactobacillus*, *Ihubacter*, *Parasutterella*, ↑ genus: *Escherichia/Shigella*, *Bilophila*, *Parabacteroides*	[113]
Humans	Whole blood, cord blood, postnatal serum and faeces	Nil	7, 14, 22 and 28 years	M, F	PFOS PFOA	Cohort study	Nil	PFOS: ↑ species: *Bilophila wadsworthia*, *Faecalibacterium prautzii*, *Dorea longicatena* and *Sutterella wadsworthensis* PFOA: ↑ species: *Bacteroidetes* and *Odoribacter splanchnicus*	[114]
Mice	Liver and faeces	4 groups of 5	8 to 10 weeks	M	PFOS	0, 0.003%, 0.006%, and 0.012%	21 days	↓ phylum: Firmicutes and Bacteroidetes ↓ genus: *Flavonifractor* and *Alistipes* ↑ phylum: Firmicutes ↑ genus: *Clostridium* and *Streptococcus*	[115]
Mice	Brain, blood, liver, intestine and faeces	5 groups of 10	8 weeks	M	PFOA	0, 0.5, 1, and 3 mg/kg	5 weeks	↓ phylum: Firmicutes, Verrucomicrobia, Actinobacteria and Bacteroidetes ↓ genus: *Ruminococcus, Anoxybacillus*, *Gemmiger*, *Akkermansia*, *Bifidobacterium* and *Parabacteroides* ↓ integrity of their intestinal barrier ↓ concentrations of SCFA ↓ capability in spatial memory and learning ↑ phylum: Bacteroidetes	[116]

Abbreviations: ↑ indicates an increase in the condition or level; ↓ indicates a decrease in the condition or level.

## 4. Acetaminophen

Acetaminophen is a non-opioid antipyretic and analgesic agent. It is also known as paracetamol and N-acetyl-para-aminophenol (APAP). Commonly used to relieve pyrexia and/or pain, acetaminophen is also used to treat sore throat pain, toothaches, headaches, backaches, menstrual cramps, and osteoarthritis pain. It can also be combined with other medicines prescribed for insomnia, cough, flu, cold, and allergies. As one of the most active and readily accessible drug ingredients, acetaminophen is found in brand names such as Tylenol, Panadol, Actamin, Feverall, Tempra Quicklets, Dayquil, and Percocet. It is recommended that users strictly adhere to the recommended dosage of acetaminophen as an overdose can be hepatotoxic and potentially fatal. Symptoms of acetaminophen intoxication include nauseousness, loss of appetite, vomiting, lethargic, sweating, yellowing of the skin or eyes, and abdominal pain in the upper right quadrant [118].

### 4.1. Autism Epidemic and Acetaminophen

In 1980, the CDC issued public warning about the possible attribution to Reye’s syndrome in children from the usage of aspirin. Since then, acetaminophen was used as the preferred medication by parents and hospitals [119]. Unfortunately, a parent survey had shown that children who were prescribed acetaminophen for fever or pain after the mandatory measle–mumps–rubella vaccine (MMR) had a higher possibility of becoming autistic as compared to children given ibuprofen [120]. By 1995, the number of children who regressed into autism at the age of about 18 months old had markedly increased to more than ten times, and children born autistic by three to four times. These increases were related to prenatal consumption of acetaminophen during pregnancy [119]. To further link the usage of acetaminophen to the increasing trend of autism in the U.S., the incidence rate was compared with that in Cuba where the children were also administered the MMR vaccine but did not use acetaminophen. At that time, acetaminophen-containing products were not readily available over the counter in Cuba, and it was only given with a doctor’s prescription. This contributed to a lower autism incidence in Cuba as compared to the U.S., with a ratio of 1:300 [119]. However, it is important to note that another possible contributing factor to the low autism incidence in Cuba was the organic agriculture industry, which discouraged the use of chemicals for pest control. Pesticides have been shown to increase the risk of autism both pre- and postnatally [121].

### 4.2. Liver Inflammation and Acetaminophen

In the liver, drug metabolizing enzymes convert acetaminophen to a reactive toxic metabolite, N-acetyl-p-benzoquinone imine (NAPQI). This metabolite requires glutathione for its detoxification, forming an acetaminophen–glutathione. At higher doses, the detoxification process depletes hepatic glutathione by as much as 80–90% and the metabolite binds covalently to proteins instead. Although some studies suggest a linear relationship between the amount of covalent binding and relative hepatoxicity, other research has shown that covalent binding may not be the main mechanism of toxicity; rather, it may be the oxidative stress from glutathione depletion that initiates the development of toxicity [122].

As glutathione stores dwindle, undetoxified NAPQIs bind to other targets, such as proteins, unsaturated lipids, DNA, and nucleophilic macromolecules, resulting in a cascade of downstream hepatocellular death events (Table 4). In addition to the decrease in mitochondrial function, studies also found that the production of ROS and glutathione disulfide (GSSG), a marker of intracellular ROS formation, was increased [123]. APAP also induced the formation of peroxynitrite in the mitochondria through the combination of a superoxide anion and nitric oxide derivatives. The formation was found to be consistent with the level of subcellular fractionation of liver tissue necrosis in mice [124]. These results suggest that an effective method to measure liver cytotoxicity may be immunohistochemistry, i.e., staining the nitrotyrosine protein adducts in the necrotic cells [125].

### 4.3. Neuroinflammation and Acetaminophen

Prenatal exposure to APAP has been reported to increase the risk of development of ADHD and ASD by 30% and 20% respectively [126]. APAP can cross the human placental barrier and disrupt the balance of endogenous hormones and many signaling pathways related to development and growth in the fetus. APAP exposure can also be neurotoxic to murine cerebrocortical neurons [127] and inhibit the production of testosterone [128], which is crucial for brain development (Table 5).

In pre-pubertal children, glutathione depletion is a contributing factor for ASD development. In children, glutathione helps convert serum adrenal androgen dehydroepiandrosterone (DHEA) into the storage form DHEA sulfate, which is a normal metabolic process. Without glutathione, DHEA becomes androstenedione and testosterone. Low DHEA sulfate levels in children are associated with functional lateralization and anatomical asymmetry in the brain [119]. For healthy fetal growth and maturation, the fetal adrenal cortex needs the essential placental estrogens that are made from DHEA sulfate. It ensures the growth of white matter with matured myelin sheaths, fatty oligodendrocytes, and nerve fibers (axons). Boys with autism and rapid brain growth in the first few years of their life were observed to have disproportionate growth between the brain hemispheres. Within hemispheres, there were larger white matter tracts, and in communications between the hemispheres, smaller white matter tracts. This implies that the sizes of the brains were bigger with disproportionately smaller corpus callosums. As interhemispheric cortical communication depends mainly on the information exchange through the corpus callosum, asymmetrical growth within the cerebrum impairs signal communication between the cerebral hemispheres [119].

Myelin water fraction, an MRI imaging biomarker for myelin, is another useful metric for tracking white matter maturation and its relationship with cognitive development in the developing brain. Healthy myelination process in the brain involves the lengthening and thickening of myelin sheaths as the axons lengthen and lipids are deposited. This process is androgen/estrogen dependent; testosterone contributes to the longitudinal growth of myelin sheaths by conversion into dihydrotestosterone, while estrogens play a significant role in the maturational growth by stimulating the depositing of lipids to displace water. Within the cerebral white matter of 6- to 12-year-old autistic boys, more water than lipids was observed using transverse relation time imaging. Other comparisons also support the finding of an immature myelin water fraction in autistic people, increased overall brain volume (larger white matter), abnormal myelination, and widespread myelin water fraction reduction, indicating a low myelin level that was associated with reduced connectivity [119].

Despite its known potential for neurotoxicity, APAP has also been reported to induce neuroprotective effects. At medically recommended dosages, APAP was able to prevent ROS-mediated neuronal cell damage and mitochondrial redox impairment. As compared to the control mice, lower levels of lipid peroxidation and calcium ATPase activity and higher levels of reduced glutathione (GSH), glutathione peroxidase, and vitamin E were observed in the brain of mice dosed with 5–100 mg/kg APAP [129]. These data suggest that, at moderate strength, APAP may have antioxidant properties to prevent oxidative stress. Other studies also supported a potential protective role by showing that APAP acts as an ROS scavenger to: (1) protect dopaminergic neurons from 1-methyl-4-phenylpyridinium-induced toxicity in mitochondria; (2) significantly reduce superoxide generation caused by quinolinic acid in the rat hippocampus; (3) increase cell survival in endothelial cell culture and inhibit the expression of SOD and inflammatory proteins that induce the superoxide-generating compound menadione; (4) limit protein oxidation by attenuating the damaging effects of hydrogen peroxide and peroxynitrite in the heart; and (5) protect hippocampal neurons as well as cells in the rat PC12 fetal neuron-like cell line from amyloid-beta peptide-induced oxidative stress by reducing phospholipid peroxidation [56,125,129,130,131,132,133].

### 4.4. Gut Microbiome and Acetaminophen

Prenatal APAP exposure is associated with low gut bacterial diversity and lasting alterations in microbiome composition in childhood. It was found that some gut bacteria could alleviate the harmful effects caused by potentially neurotoxic compounds, such as APAP, and contribute to their metabolism [134]. For most gut microbiome sampling, the feces are retrieved for analysis. However, in the fetus where most compounds undergo metabolism through the placenta, and in fetal liver by the third trimester, the meconium is the ideal matrix for analysis as metabolized compounds would accumulate there. In children with prenatal exposure to APAP, it was observed that higher concentrations of Proteobacteria were associated with lower Wechsler Intelligence Scale for Children (WISC)-IV subscales [135]. Through clinical, epidemiology, and animal studies, an elevated relative abundance of Proteobacteria, specifically Gammaproteobacteria, which includes notorious pathogens such as Shigella, Escherichia, and Salmonella, is associated with cognitive impairment and low cognitive performance, such as general knowledge and short-term memory [135].

As mentioned in the section above, an overdose of APAP causes dysbiosis in the gut and impairs sulfate metabolism, which would eventually lead to neuroinflammation. Within the gut, anaerobic fermentation takes place and produces ammonia and short chain fatty acids. These processes may cause an overgrowth of anaerobic bacteria such as Bacteriodetes, Clostridia, and Desulovibrio, which in turn cause a decrease in methionine, an important antioxidant for the brain and liver [119]. Decreased methionine levels would reduce its catabolite S-adenosylmethionine and result in a subsequent loss of histone methylation [136]. As methylation impairment was associated with autism, it would be reasonable to determine if APAP exacerbates autism through epigenetic mechanisms.

**Table 5:** Studies reporting the neurotoxicity and protective effects of APAP exposure.

Organism	Sample Type	Sample Size	Age	Sex	Reagents	Exposure Dose	Exposure Time	Effects	Outcome	References
Rats	Brain endothelial cells	Not indicated	Not indicated	Not indicated	APAP	100 μM	8 h	Protective	↓ SOD activity ↓ Inflammatory proteins ↓ Superoxide-generating compound menadione ↑ Cell survival	[56]
Rats	Plasma and cerebrospinal fluid	2 groups of 6–8	Not indicated	F	APAP	250 and 500 mg/kg	1, 3 and 6 h	Toxic	↑ Neuronal death in cortex	[127]
Rats	Testes (ex vivo organotypic culture)	Not indicated	Gestation day 14.5	M	APAP	0.1 μM to 100 Μm	24, 48 and 72 h	Toxic	↓ Testoesterone production ↓ Brain development	[128]
Rats	Brain	70	Adult	M	APAP	5 to 100 mg/kg	24 h	Protective	↓ ROS ↓ Mitochondrial redox impairment ↓ Lipid peroxidation level ↓ Ca2+- ATPase activity ↑ Reduced glutathione (GSH) ↑ Glutathione peroxidase (GSH-Px) ↑ Vitamin E	[129]
Rats	Hippocampal neurons and PC12 cell line	Not indicated	Not relevant	Not relevant	APAP	10 mM	24 h	Protective	↓ Lipid peroxidation level ↓ Amyloid-beta peptide-induced oxidative stress	[130]
Guinea pigs	Heart	2 groups of 4	Not indicated	M	APAP	0.35 mM	1 time	Protective	↓ Protein oxidation ↓ Damaging effects of hydrogen peroxide and peroxynitrite in the heart	[131]
Rats	Brain	24	Adult	M	APAP	100 mg/kg	3 h (injection/hour)	Protective	↓ 1-methyl-4-phenylpyridinium induced toxicity in mitochondria; Protected dopaminergic neurons	[132]
Rats	Brain (hippocampus)	5 groups of 5	Adult	M	APAP	100 mg/kg	7 days	Protective	↓ Superoxide generation caused by quinolinic acid	[133]
Rats	Whole brain Frontal cortex and hippocampal proteins	60	Adult	M	APAP	200 mg/kg	1, 15 and 30 days	Toxic	↑ Brain-derived neurotrophic factor (BDNF) expression ↓ Learning and memory (cognitive impairment)	[137]

Abbreviations: ↑ indicates an increase in the condition or level; ↓ indicates a decrease in the condition or level.

## 5. Potential Interactions between PFAS and Acetaminophen in Exacerbating Autism Spectrum Disorder

Liver inflammation is the hallmark of PFAS and acetaminophen toxicity, and studies had shown comparable symptoms induced by both chemicals through oxidative stress, a reduction in glutathione levels, and cell necrosis [80,86,89,123,124]. However, numerous studies that investigated prenatal exposure to PFAS and acetaminophen have revealed disparate trends for changes in the microbial community, suggesting that these two chemicals may induce different effects on the gut microbiota [10,110,114,115]. Unfortunately, their synergistic impacts on health have not been studied. Our hypothesis posits that, under acute exposures, acetaminophen has the ability to aggravate the impaired conditions of the PFAS-exposed liver, which would further exacerbate neurological symptoms such as lack of social communication and interest, and repetitive behaviors using mechanisms related to the gut–liver–brain axis.

Studies with a focus on the gut–liver–brain axis will be required for a clearer view on how PFAS and acetaminophen interactions might contribute to autism in humans. Within the gut–brain axis, an impaired gastrointestinal homeostasis (e.g., SCFAs, microbiome, tryptophan, indole, bile acids) could disrupt the expression of BDNF, the production of endocrine signals (e.g., GLP-1), and neurotransmitters (e.g., GABA and serotonin). These perturbations, in turn, may lead to the impairment of normal brain development including that of microglia, the primary form of active immune defense in the CNS. Within the gut–liver axis, gut dysbiosis might induce a reduction in essential receptors, e.g., TGR5 and FXR, which would lead to a cascade of downstream alterations such as a decrease in secondary bile acids synthesis, followed by the retention of bile salt, bacterial overgrowth, liver disease and, in worse scenarios, liver damage. Within the liver–brain axis, CNS dysfunction could potentially arise from an altered blood flow, presence of inflammatory metabolites and excess bile acids, and accumulation of neurotoxic compounds. The onset of liver injury and inflammation might lead to a systemic elevation of proinflammatory cytokines (IL-6, IL-1 β, TNF-α), which in turn would affect peripheral neural signaling and compromise the blood–brain barrier, thereby impacting neuronal function. NAFLD has also been briefly linked to ASD, due to the common finding of nuclear inclusions in hepatocytes found in people with liver diseases and BTBR mice, a model of ASD [138]. Moreover, recent studies have shown that the left vagus nerve is the carrier of signals from the liver to the brain [74]. As the vagus nerve is a newly discovered neuroimmune pathway, studies exploring the interaction between PFAS and acetaminophen through the vagus nerve within the gut–liver–brain axis would be beneficial in the future.

Through exposure and epidemiological studies, there was evidence that PFAS and acetaminophen could potentially trigger neurobehavioral disorders [93,126]. Three mechanisms (i.e., calcium homeostasis, calcium-dependent signaling molecules, and neurotransmitters) have been useful in explaining the exacerbation of neurological diseases by PFAS and, possibly acetaminophen. In the case of acetaminophen, glutathione depletion is a good indicator to determine if pre-pubertal children may regress into autism, as it would eventually contribute to functional lateralization and anatomical asymmetry in the brain [114]. Glutathione depletion has also been useful in assessing hepatoxicity induced by PFAS. As both chemicals have been hypothesized to cause neurological disorders, myelin water fraction is another useful metric to track white matter maturation and its relationship with cognitive development in the developing brain. A disrupted growth of white matter and myelination in the brain might cause a disproportionate ratio between the brain and its corpus callosum, which would impact the communication between the hemispheres [119].

BDNF is a protein critical for learning and memory and is mostly expressed in the CNS and gastrointestinal tract. The expression of BDNF also indicates the level of cognitive impairment, and this biomarker is reliable for acetaminophen neurotoxicity [137]. Recent studies in which rats received a prolonged APAP treatment found strong associations between the expression of BDNF and the level of cognitive impairment [137]. However, it is unclear if BDNF is reliable for most PFAS due to the impenetrable blood–brain barrier [117], as the mechanisms of PFAS transportation into the blood–brain barrier remain unverified. Studies of PFAS exposure using animal models found a strong association between the concentration of PFHxS and BDNF expression, but these were unable to conclude if BDNF was a reliable biomarker to measure neurodevelopment as there were no associations for other PFAS [117].

## 6. Conclusions and Future Directions

The globally rising prevalence of autism and PFAS exposure suggest a need to explore a possible causative association that takes into account the toxic effects transmitted through the gut–liver–brain axis more thoroughly. Although many studies have been conducted in terms of acetaminophen liver toxicity, the molecular and cellular mechanisms remained inadequately elucidated. However, some could be inferred by following the mechanisms elicited from PFAS hepatoxicity, such as the association of biomarkers (ALT, ALP, AST, and GGT), receptors (PPARα, CAR, and PXR), inflammatory markers (TNF-α and IL-6), unfolded protein response signaling pathway markers (IRE1α, ATF4, and BIP), fatty acid metabolic gene markers (SCD1, FASN, ACC, and SREBP1), fibrosis signaling gene markers (TIMP2, p21, and TGFβ), and gene expressions associated with NAFLD. The bidirectional relationship between the liver and gut is becoming increasingly apparent, verifying the need for studies encompassing alterations in these interconnections. The interactions between PFOS/PFOA and gut microbiota are well-represented in the literature, however, there is a lack of information about co-exposure to agents like acetaminophen. As the well-known liver toxicant acetaminophen was found to contribute to the risk of autism [119,126], this review advocates for more studies to determine potential interactions between PFAS and acetaminophen.

The potential interactions between PFAS and acetaminophen discussed in the early section would provide some perspectives into the provocative question—“Does use of acetaminophen combined with PFAS exposure synergistically increase risk of autism?” Combined epidemiological and animal studies will again be needed to provide more definite answers. Furthermore, the manifestations of harmful synergistic effects will be dependent on various factors, which include doses/exposures, dosing period, and the genetic profile of individuals. For example, it remains an open question whether a multiple dose regimen of acetaminophen at moderate strength could elicit neurotoxic effects, while a single moderate-strength dose may induce neuroprotective effects in mice [125,129,137]. Collective observations summarized in this review underscore several considerations for future research directions in the exploration of potential mechanisms underpinning neurotoxicity resulting from the interactions between PFAS and acetaminophen. These include, but are not limited to: calcium homeostasis, calcium-dependent signaling molecules, neurotransmitters, glutathione depletion, oxidative stress, cell necrosis, disruption or transporter binding at the blood–brain barriers, disrupted growth of white matter and myelination in the brain, vagus nerve signaling and the association of inflammatory, related signaling pathways, and metabolic gene markers. In addition, more can be done to establish a concrete gut–liver–brain axis in the intricate interactions between acetaminophen and PFAS, which would, ultimately, serve as a guided roadmap for researchers to uncover more underlying mechanisms and pathways of various diseases linked to this axis. In particular, in the study of autism, neurotoxicity research should be prioritized to determine the downstream pathways relating to the gut and liver axis. Recent research has identified the vagus nerve as the neuronal pathway for gut–liver–brain interactions [43,44,74,75]. However, the studies only focused on the transmission of signals from the gut microbiome to the CNS through the vagus nerve, leaving the potential involvement of the vagus nerve, directly or indirectly, in playing a part in PFAS or acetaminophen neurotoxicity via the gut–liver–brain axis unexplored.

By strengthening the research on the gut–brain–liver axis, extending beyond the scope of PFAS, acetaminophen, and autism, this future direction would yield deeper insights into the relationship between various targeted chemicals and diseases. The axis can also contribute to the adverse outcome networks, where evidence-based succession of multilevel key events link to outcomes from different individual adverse outcome pathways (AOP). By connecting neurologically aggravated mechanistic pathways to the extensive AOP network, chemicals that promote neurological disorders and symptoms could be identified. In this way, regulatory actions can be called upon to limit the use of neurotoxic chemicals, and the risk of autism due to environmental factors would eventually be reduced.

## Figures and Tables

**Table 4:** Studies reporting the liver toxicity effects of APAP exposure.

Organism	Sample Type	Sample Size	Age	Sex	Reagents	Exposure Dose	Exposure Time	Outcome	References
Mice	Liver cells	Not indicated	Not indicated	M	APAP	5 mM	0 to 12 h	↓ Mitochondria function ↑ ROS production ↑ Glutathuone disulfide (GSSG)	[123]
Mice	Plasma, blood, and liver	5 groups of 4–6	Not indicated	M	APAP	300 mg/kg	12 h	↑ Peroxynitrite in mitochondria ↑ Liver tissue necrosis	[124]

Abbreviations: ↑ indicates an increase in the condition or level; ↓ indicates a decrease in the condition or level.

## Data Availability

Not applicable.
